# Community Engagement for Big Epidemiology: Deliberative Democracy as a Tool

**DOI:** 10.3390/jpm4040459

**Published:** 2014-11-20

**Authors:** Rebekah E. McWhirter, Christine R. Critchley, Dianne Nicol, Don Chalmers, Tess Whitton, Margaret Otlowski, Michael M. Burgess, Joanne L. Dickinson

**Affiliations:** 1 Menzies Institute for Medical Research, University of Tasmania, Hobart TAS 7000, Australia; E-Mails: Rebekah.McWhirter@utas.edu.au (R.E.M.); Dianne.Nicol@utas.edu.au (D.N.); Don.Chalmers@utas.edu.au (D.C.); 2 Centre for Law and Genetics, Faculty of Law, University of Tasmania, Hobart TAS 7001, Australia; E-Mails: ccritchley@swin.edu.au (C.R.C.); Margaret.Otlowski@utas.edu.au (M.O.); 3 Faculty of Health, Arts and Design, Swinburne University, Melbourne VIC 3122, Australia; 4 W. Maurice Young Centre for Applied Ethics, University of British Columbia, Vancouver, BC V6T 1Z2, Canada; E-Mail: Michael.burgess@ubc.ca

**Keywords:** community consultation, public engagement, big epidemiology, personalized medicine, biobanks, ethics, deliberative democracy

## Abstract

Public trust is critical in any project requiring significant public support, both in monetary terms and to encourage participation. The research community has widely recognized the centrality of public trust, garnered through community consultation, to the success of large-scale epidemiology. This paper examines the potential utility of the deliberative democracy methodology within the public health research setting. A deliberative democracy event was undertaken in Tasmania, Australia, as part of a wider program of community consultation regarding the potential development of a Tasmanian Biobank. Twenty-five Tasmanians of diverse backgrounds participated in two weekends of deliberation; involving elements of information gathering; discussion; identification of issues and formation of group resolutions. Participants demonstrated strong support for a Tasmanian Biobank and their deliberations resulted in specific proposals in relation to consent; privacy; return of results; governance; funding; and, commercialization and benefit sharing. They exhibited a high degree of satisfaction with the event, and confidence in the outcomes. Deliberative democracy methodology is a useful tool for community engagement that addresses some of the limitations of traditional consultation methods.

## 1. Introduction

### 1.1. Why Is There a Demand for New Methodology in Community Engagement?

A key development in the era of personalized medicine is the ability to dissect the interactions of lifestyle and environmental determinants of disease incidence, disease course and treatment response [1], and to explore their association with genetic background and biochemical markers. Increasingly, medical researchers rely on biobanks to provide tissue and information to facilitate their research. The potential of such resources has led to a proliferation of consortia, distributed data networks, large-scale data linkage units and biobanks that collect data and biological samples from large, population-based, prospective cohorts, enabling multi-centre and cross-disciplinary research [2,3,4]. New ethical, legal and methodological issues have arisen alongside establishment of these resources, relating to consent, privacy, return of results, governance, funding, commercialization and benefit sharing [5,6,7]. The success of these ventures is therefore highly dependent on the trust and goodwill of the populations from which resources are drawn. Widespread public support is necessary to achieve viable participation rates from unwell and healthy cohorts within the population, and to secure adequate financial support. This is particularly evident in prospective longitudinal studies, which rely on positive community engagement and lasting public trust for adequate participation and retention rates [8].

One of the cornerstones of medical research in the personalized medicine age is the generation of big data. “Big” epidemiology can encompass a range of large studies such as those with large numbers of participants (e.g., 100,000 or more) or those that require storage and analysis of massive amounts of genomic data [9,10,11,12]. An integrated population data resource for large-scale population research would provide a source of big data, ripe for use in very big epidemiological studies. The caveat to this is that the success of creating and maintaining such a resource relies upon gaining and retaining public trust.

The government of Iceland, in partnership with deCODE Inc., created a “national genomic databank” [13], full of this promise and expectation. But the government’s sale of individual medical information to a private enterprise—without consent from biobank participants—resulted in the failure in 2003 of Iceland’s Health Sector Database (HSD) [14]. The HSD provides a high-profile example of the need for community consultation and public engagement that is responsive to societal attitudes during planning of large repositories and beyond [13,14,15]. Since then, public concerns regarding privacy, genetic discrimination and stigmatization have continued to grow, further cementing the need to integrate community partnerships into research [16,17]. The community consultation undertaken for the Iceland HSD and also for the Estonian Genome Project utilized a “communication type of approach”, based on public education and quantitative assessments of public opinion [18,19]. In subsequent efforts, the UK Biobank, Generation Scotland and CARTaGENE in Quebec, have employed more of a “partnership approach” [18,20]. Aimed at facilitating open dialogue between researchers and community members, through utilization of both quantitative (surveys) and qualitative (focus groups and interviews) methods [15,18,19,20], these approaches recognize that community members may identify risks and formulate safeguards that are more acceptable to the community than those developed by researchers. This is especially relevant where moral, cultural or spiritual frameworks differ, such as when indigenous populations are included.

One criticism that has been leveled at these more participatory approaches is that there tend to be deficiencies in the consultative process, including restrictions on the scope of discussion to certain topics, and focus on garnering support for the project design rather than engaging in genuine consultation to improve it [21,22,23]. A key requirement in community engagement is to ensure the consultation goes beyond providing information and reassuring participants, to include respectful, genuine debate and dialogue between researchers and members of the public.

Traditional surveys, focus groups and interviews, whilst useful, have recognized limitations particularly when the subject matter is complex or unfamiliar, requiring iterative dialogue to address these shortfalls [24,25]. In the case of big epidemiology where community members are unfamiliar with concepts like data linkage or biobanking, these traditional methods are likely to result in “top of the head” reactions, which may not assist policy formation or study design [24]. In these situations, traditional consultation techniques are poor tools for involving the public in setting the agenda, debating issues in an informed manner, and contributing to the resolution of concerns.

Community consultation and engagement in the study design phase of a research project has long been common in research involving indigenous populations and particularly in genetic research, but is becoming more widely employed [26,27]. Further, many funding bodies include community engagement in their assessment criteria and require reporting against these measures. Goals of community consultation include: identifying potential problems with the study design; improving public scientific literacy; assessing community priorities for research agendas; and improving participation rates. Community consultation also provides accountability for science and has the potential to build public trust [24].

### 1.2. What is Deliberative Democracy?

Within the context of the present study, “deliberative democracy” is used to describe a method for community engagement in complex ethical issues surrounding emerging technologies, stemming (but distinct) from the political theory of the same name. This method is distinguished from focus groups by the purposeful provision of information to participants, and by the emphasis on dialogue leading to iterative revision of opinions by the participants as they integrate new information and others’ perspectives [28]. Public participation with two-way dialogue between researchers and members of the public has been employed in a variety of settings. Deliberative democracy forums have been employed to facilitate informed and meaningful public input into a range of biotechnology issues, including biobanking, in Canada, the US and Australia [29,30,31,32]. Although this term is loosely used to cover a variety of consultation methods including citizens’ juries, consensus conferences and deliberative polls [33], it refers also to a more structured process of community consultation, such as the event described here [34,35]. Deliberative democracy events have demonstrated utility in a number of contexts, including public health settings, such as population screening in cancer [1,36]. To date, this methodology has not been employed extensively and it has a wider potential application in big epidemiology and biobanking.

In the policy context relevant to the present study, although the Australian National Health and Medical Research Council has developed a National Biobanking Strategy [37], there remain no national guidelines for the development, operation and governance of biobanks in Australia. Thus, many of the ethical issues associated with privacy, return of results, broad consent and commercialization (among others) remain unresolved. A community consultation model built around the concept of deliberative democracy was therefore implemented in Tasmania, the island state of Australia, as part of a larger consultation project [38]. Herein we outline the methodology employed in the deliberative democracy event that formed a key component of this consultation process, and provide an evaluation of its potential within the broader epidemiological research context. This consultation was undertaken to ascertain community attitudes towards biobanking and to provide members of the Tasmanian public with an opportunity to contribute towards developing a governance framework.

### 1.3. Why Tasmania?

Tasmania has proven to be a rich resource for both genetic and epidemiological studies [39,40,41,42]. Productive research has been facilitated by detailed genealogical records, good health care services, and a willing population, together with logistical benefits resulting from relatively small geographic distances and small numbers of health care providers for ease of data collection. These conditions suggest that Tasmania would be an appropriate place for a biobank to be established. Coincidentally, and equally importantly for our study, these factors provide an ideal environment to engage in deliberative democracy in Tasmania, to guide future development of governance and management frameworks for large-scale biobanking and epidemiological studies.

The purpose of this event was to engage the Tasmanian community in dialogue regarding the potential establishment of a state-wide Tasmanian biobank.

## 2. Methods

Ethical approval for this project was received from the Tasmanian Human Research Ethics Committee.

### 2.1. Recruitment

The aim was to recruit 25 members of the Tasmanian public to represent a diversity of public views relating to biobanking using demographic characteristics as a proxy for different views. Recruitment strategies aimed to avoid selecting individuals with vested interests, which commonly skews community engagement efforts, and to target generally underrepresented groups [34], including indigenous people and those from low socio-economic backgrounds. Potential participants were initially contacted through computer-assisted telephone interviews, using randomly selected numbers from publicly-listed Tasmanian residential telephone numbers. A short interview script informed potential participants about the event, gained their interest (or not) in participating, and collected information on their background characteristics (see Supplemental Materials S1). It was delivered by four trained telephone interviewers, supervised by one of the authors. All Tasmanian residents over the age of 18 years were eligible to take part in the event. To ensure comparability with international events on biobanking, the demographic quotas of Longstaff and Burgess [34] were adapted to the local population (see Table 1). All participant expenses were paid and they received a $100 stipend per day of attendance across two weekends (*i.e.*, 4 days), which largely followed the design of the earlier Canadian events [35]. All participants gave informed consent.

**Table 1:** Required and achieved numbers of participants across demographic categories for all stages of recruitment and participation.

Demographic		Required		Achieved Stage 1		Achieved Stage 2		Selected		Participated in Event	
Characteristic	Category	F	M	F	M	F	M	F	M	F	M
Location	Hobart	2	2	19	9	6	4	9	5	8	3
	Other towns	2	2	13	9	1	1	5	7	4	5
	Rural	2	2	21	7	0	0	5	5	4	1
Age	≤30 years	1	1	3	1	1	4	4	2	3	1
	>30 years	1	1	50	24	6	1	15	15	13	8
Education	No university	1	1	38	18	5	5	14	12	12	5
	University	1	1	15	7	2	0	5	5	4	4
Employed	Yes	1	1	17	8	7	3	11	11	9	6
	No	1	1	36	17	0	2	8	6	7	3
Born in Australia	Yes	1	1	40	21	6	3	13	12	11	6
	No	1	1	13	4	1	2	6	5	5	3
Indigenous	Yes	1	1	2	1	0	0	2	1	2	0
	No	1	1	51	24	7	5	17	16	14	9
Genetic or serious medical condition	Yes	1	1	24	14	0	0	7	10	7	5
	No	1	1	29	11	7	5	12	7	9	4

One person may represent more than one demographic category; F = female, M = male.

A summary of the recruitment strategies and metrics is presented in Table 1. Of 554 calls that resulted in communication with a household member, 75 expressed an interest in attending, 473 declined and one respondent was unsure. Of those who declined, nine agreed to receive further information by post, and after being re-contacted by telephone, three of these agreed to participate. The cohort of 78 respondents exhibited overrepresentation of females and older people, and underrepresentation of employed individuals and people from the Hobart district (the largest city in the state). There were also some categories with small numbers (notably Indigenous and young male participants) that were at risk of unacceptable attrition in the lead up to the event. A second round of telephone interviews targeted potential participants in these at-risk categories. A total of 927 screened calls (to identify Indigenous people and young males) were made within the Hobart region, of which 236 resulted in speaking to a household member, and an additional 12 people agreeing to participate.

Of the thirty-six participants that were selected for the event, eight withdrew ahead of the event and three failed to attend on the first day, resulting in 25 participants, as targeted, attending and completing the event.

### 2.2. Information Provision

Deliberative democracy requires that participants possess a degree of knowledge about the issues, to give their discussions meaning and legitimacy in a policy context [43]. Unduly influencing the participants or framing the debate (either intentionally or otherwise) in a particular way is a recognized risk [22]. To mitigate this risk, the research team consulted widely and invited contributions from a broad range of stakeholders when developing information materials. Information was provided to participants in a variety of formats to account for variations in learning styles, and included:

- Prior to the event
  (i) an information booklet mailed to participants (detailed [44]);
  (ii) a website (with links to relevant external websites);
  (iii) an invitation to participants to independently seek out information, and to discuss issues with their friends and family.
- During the event
  (i) informative presentations by five expert speakers representing the diverse perspectives of researchers (what is a biobank and the value of biobanks to genetic research), lawyers (presenting issues of privacy, consent and conflicts of interest), the indigenous community (autonomy and ethical issues) and those affected with a genetic disease (autonomy, genetic discrimination and other ethical issues); and
  (ii) a panel Q&A session, which included end users such as a representative from the Department of Health and Ageing and experts in genetics, law and biobanking [45].

A total of 10 (43.5%) participants indicated that they had engaged in some independent research on biobanks before the event, and 13 (54.2%) conducted some research during the break between the first and second weekend.

### 2.3. Small and Large Group Format

**Figure 1:**
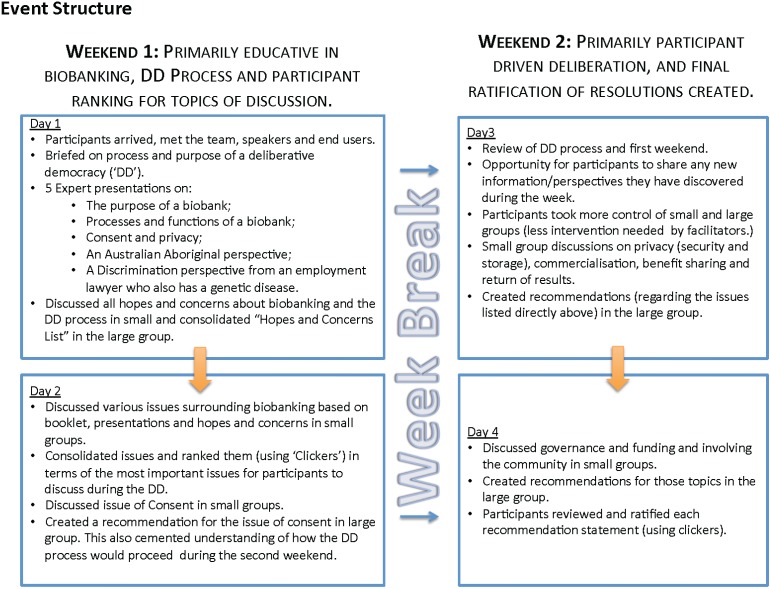
Content and structure of the deliberative democracy event.

Each day was divided into short time allotments, comprising a mix of the following activities: informative presentations; small group discussions; large group discussions; Q&A panels; voting; and resolution formation (see Figure 1) [24]. Small allocated groups of 8–10 participants were facilitated by members of the project team and functioned as think-tanks, allowing story-telling and sharing of ideas, enabling participants to identify their key hopes and concerns. Small group discussions provided space for all participants to express their opinions in a supportive environment; notably, even participants who were reluctant to contribute to large group discussions felt comfortable participating in the small group format. In sharing their differing perspectives, participants were encouraged to engage with each other’s positions and to consider revising their own in light of new information or novel perspectives. Group consensus was encouraged but not enforced; one of the strengths of this approach is that areas of disagreement that persist even after considered and informed discussion can be identified and taken into account when developing policy. Ideas from the small groups were relayed during discussions in the large group. Large group discussions, led by the facilitator (MMB) aimed to resolve areas of contention. Topics developed as participants identified new priorities. An electronic audience response system (TurningPoint Software, Keepad Interactive), “clickers”, was used to aggregate results and permit identification of areas of persistent disagreement. The information booklet was also used as a tool for discussions in the small groups when participants required an aid to prompt discussions.

### 2.4. End Users and Observers

The event was designed primarily to gauge public perceptions of biobanking and inform policy. People in positions to influence policy and practice were invited to attend as observers, such as representatives from the National Health and Medical Research Council, Department of Health and Ageing and the Tasmanian Data Linkage Unit. Those attending in these capacities were available for the Q&A interactive large group sessions with the participants. These professionals directly observed and participated in the event, and have been able to incorporate participant recommendations into future policy decisions.

### 2.5. Participant Satisfaction Surveys

Participants were given an identical satisfaction survey at the conclusion of each weekend’s proceedings (see Supplemental Materials S2). This survey comprised a range of questions designed to assess participants’ perspectives on the event’s structure and presentation, the outcomes reached, and satisfaction with the event as a whole. It also assessed the level of trust participants felt in the group processes and outcomes, and intention to participate in such an event again.

### 2.6. Statistical Analyses Method

All statistical analyses were undertaken in SPSS v. 21 (Chicago, IL, USA). Binary logistic regressions were used to investigate factors influencing withdrawal from the event. Participant satisfaction survey responses were summarized using proportions (for yes/no responses) or medians and ranges for ordinal variables.

## 3. Results

### 3.1. Participants

Of the 36 individuals who were invited to attend the event, 25 attended in full, giving an attrition rate of 30.6 percent (11/36). Of the 25 attendees, there were 16 females and nine males, with only the Indigenous male category unrepresented. A series of logistic regressions predicting drop out status revealed that males were 79 percent more likely to withdraw than females (*p* < 0.05). Withdrawal was not associated with age, country of birth, education, experience of a genetic or serious illness, location or employment status, but results should be interpreted with caution due to the small sample sizes.

### 3.2. Outcomes

**Figure 2:**
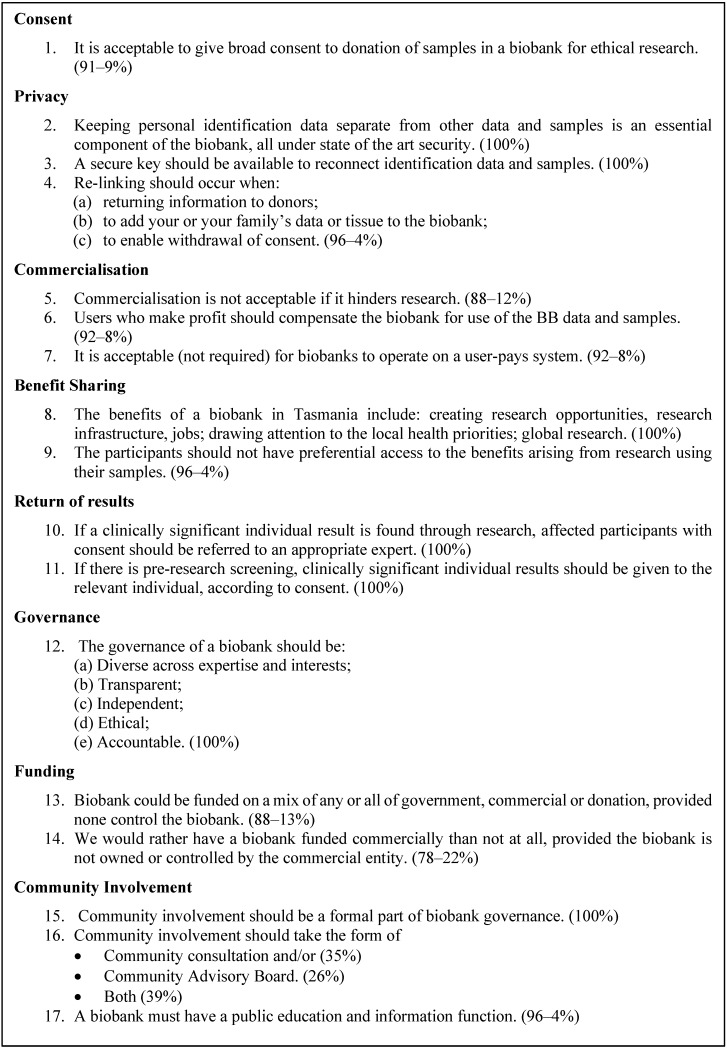
Participant outcomes with large group ratification results (Agree-Disagree%).

During the course of the deliberation, the participants identified and refined their collective hopes and concerns regarding biobanking. These were used to guide discussion, leading to the identification of issues that were important to them and, ultimately, create resolutions outlining their considered opinions regarding each of the identified issues (see Figure 2). Governance is an issue that is frequently highlighted in relation to biobanks [46], and this is reflected in several of the group’s resolutions. However, this cohort particularly focused on security and storage procedures, which were discussed at length. For example, the process by which a sample might be re-identified from its de-identified form was considered in depth, with particular focus on the following: who should have access to this information (two people would have keys to re-identify); and where it should be stored, geographically (in two places in case of natural disasters) and technically (identified information was to be stored on a computer with no remote access). Although academics generally consider these topics as part of procedures of governance and privacy, and the research team expected them to be discussed in that manner, participants categorized these issues separately and re-prioritized them according to their high level of concern. This led to the resolution: “A secure key should be available to reconnect identification data and samples (100% participant agreement)”.

Other key participant outcomes highlighted the pragmatism of participants in that they were generally willing to compromise on their concerns to some extent, if it would facilitate more practical outcomes. All participants bar two found it acceptable for donors to a biobank to donate via a mechanism of broad consent. This was a new outcome for deliberative democracies concerning biobanking [35,46]. Such rationality also manifested in participant views towards commercialization. Again the full group felt that although it was a real and troubling concern, commercialization may be acceptable where it is necessary to promote medical research. It seems that trust is the defining feature for participants in allowing for the aforementioned concessions (for a more detailed list of outcomes see Figure 2). Although trust relationships are complex to develop and perhaps easier to undermine, participants also indicated that trust would be greatly enhanced through formal community involvement in biobank government structures.

### 3.3. Participant Satisfaction Survey

**Figure 3:**
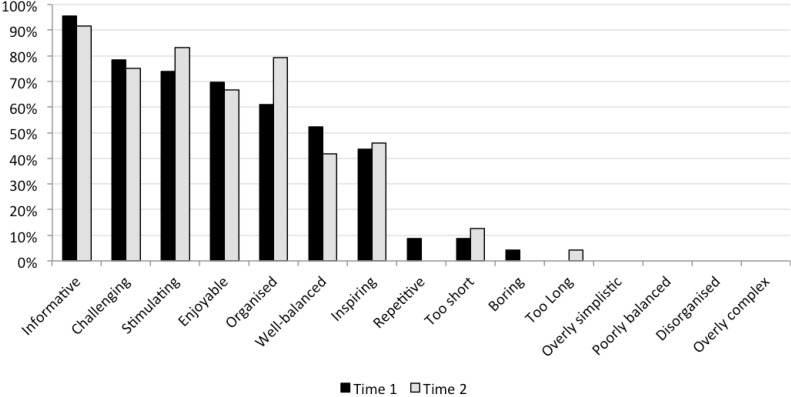
Percentages of participants selecting each attribute that described their feelings about the event.

Note: At Time 1, *n* = 23 and at Time 2, *n* = 24.

Results of the two participant perception surveys of the event are summarized in Table 2. Differences between time points were minimal, albeit with a minor trend towards more positive responses at Time 2 (at the end of the event). Consistently high median scores for all responses indicated participants overall felt respected and listened to, and trusted the event’s processes. They also indicated a willingness to abide by the group’s final position and believed that the final recommendations addressed all important issues. An overwhelming majority (*i.e.*, over 90 percent) believed it is sensible for researchers to rely on deliberative events when trying to develop policy. Most participants (over 60 percent) described the event as informative, challenging, stimulating, enjoyable, and organized. These results suggest, on the whole, that participants felt positively about all aspects of the deliberation, though they found the experience challenging. See Figure 3.

**Table 2:** Descriptive statistics for the participant satisfaction survey at Time 1 and Time 2.

Trust	Time 1		Time 2	
	Median	Range	Median	Range
Did you feel heard/listened to during the deliberation? ^a^	4	(3–4)	4	(4–4)
Did you feel respected during the deliberation? ^a^	4	(3–4)	4	(3–4)
Were the processes that led to the group’s recommendations fair? ^a^	4	(2–4)	4	(3–4)
Were the processes that led to the group’s recommendations trustworthy? ^a^	4	(3–4)	4	(3–4)
In your opinion, is it sensible for researchers to rely on a deliberative event like this when trying to develop Australian policy? ^e^	95.7% Yes	4.3% No	91.7% Yes	4.2% No/ 4.2% DK
Outcomes				
How willing are you to abide by the group’s final position, even if you personally hold a different view? ^a^	4	(2–4)	4	(3–4)
The group’s final recommendations addressed all issues considered important by participant s ^c^	3	(1–4)	3	(1–4)
Intentions and Evaluation				
How likely is it that you would attend an event like this again in the future? ^d^	4	(3–4)	4	(3–4)
How would you rate this deliberative event overall? ^b^	5	(4–5)	5	(4–5)

^a^ = scale for this item was: 1 = Not at all, 2 = A little, 3 = Somewhat, 4 = Very. ^b^ = Scale for this item was: 1 = Very poor, 2 = Somewhat poor, 3 = Neutral, 4 = Good, 5 = Excellent in response to the question, “Please rate each of the following aspects of the deliberative event”. ^c^ = scale for this item was: 1 = Strongly disagree, 2 = Disagree, 3 = Agree, 4 = Strongly agree in response to, “Please indicate your level of agreement with the following statement” (3 respondents who answered “don’t know” at time 1 were excluded). ^d^ = scale for this item was: 1 = Not at all likely, 2 = Unlikely, 3 = Likely, 4 = Very Likely (one respondent at time 2 for likelihood of attending answered “don’t know” and was excluded). ^e^ = scale for this item was: 1 = Yes, 2 = No, 3 = Don’t Know. N’s ranged from 23–24 with two participants not completing the survey at Time 1 and one at Time 2.

## 4. Discussion

There has been a paradigm shift in the way we approach studies into the causative factors for common diseases, particularly with large scale population based studies that collect large volumes of epidemiological, medical and biological samples. In parallel, we are seeing significant changes in the ethical, legal and social landscape surrounding the collection, storage, use and sharing of tissue and data arising from these large, often very costly, studies. But controversy continues to cast a long shadow over many key features of these large scale studies. For example, the ethical guidelines (in Australia, the *National Statement on Ethical Conduct in Human Research*) currently provide little guidance regarding the return of results within the context of biobanking, with the current advice aimed primarily at more traditional study designs that are more limited in size, scope and duration. Departure from this traditional model is not simple, requiring public support for a change of this significance. The vigorous debates that have ensued have resulted in calls to more effectively engage the public in informed deliberation.

Deliberative democracy is a useful tool to facilitate public engagement [45], used alone or in conjunction with other techniques, such as surveys and focus groups [20]. It surpasses other forms of public consultation because of its potential to develop trust in the process itself and in its outcomes, as demonstrated by the findings of the participant satisfaction survey. Despite the diversity in their background, nearly all participants felt heard, respected and expressed confidence in the overall decisions made by the group. They consistently emphasized the importance of community involvement. Participants generally thought it should be a formal part of biobank governance and that members of the public should not only be educated but also openly engaged with. The high levels of trust demonstrated amongst the participants in this event may therefore have been due to a perception that the researchers were committed to engage respectfully with a diverse and possibly critical group of the public.

Public trust is an essential component in any project requiring public support both in monetary terms and to encourage participation. Deliberative democracy distinguishes itself from other forms of public consultation by facilitating informed discussion, through the provision of information from a range of sources. The creation of a culture of freedom to comment and engage is essential, and all perspectives are encouraged. As such, the process facilitates dialogue between participants. Every participant spoke, of his or her own accord, at some point during the Tasmanian event. The research team noted that a benefit of having both small and large discussion groups was that perspectives of participants who did not feel comfortable speaking in the large groups were noted by more confident members of their small groups and communicated to the full group.

Deliberative democracy events of this nature culminate with identification of points of resolution and recurring disagreement, providing invaluable information, from the many voices within society, for policy and procedure formation. The presence of end-users, particularly those in positions of power regarding policy formation and implementation, is important in generating trust as their presence validates the idea that participants’ views are important and have some consequence.

It is important to note that this method of consultation is resource intensive. The Tasmanian deliberation was a product of a 14-member team that included academics in law, social science, and genetics as well as event coordinators, research assistants and recruiters. Moreover the process took a substantial amount of lead-time and was relatively expensive compared with other forms of consultation. Nevertheless, the investment of time and resources produced an event that was successful in terms of participant experience, as demonstrated by the results of the participant satisfaction survey (particularly see Figure 3). Furthermore, it enhanced the legitimacy of the consultative findings and in turn provides an important foundation for public trust.

The deliberative democracy event reported here sought to capture the geographical and social diversity of Tasmania. The aim was not to achieve a randomly selected, unbiased sample, one of the mainstays of epidemiological research, but rather to hear varied Tasmanian voices. A similar deliberative democracy event on biobanking was conducted in Western Australia and informed our deliberation [31,47,48]. However, that event reported a “lack of socio-demographic diversity in our participants” [47], and it was in order to address this limitation that the recruitment strategy employed in the current study was devised. There are significant challenges in achieving sufficient representation from rural and urban communities, ethnic and social groups and younger and older generations. Hence, every person is important, as they are each chosen for their specific attributes. Once recruited, it is vital that dropouts prior to the event and between weekends be kept to a minimum. While there were some dropouts following recruitment in this study, particularly amongst male participants, a diverse sample was maintained and there were no withdrawals once the event commenced, notwithstanding significant travel requirements for some participants. This commitment does not seem fully explained by the $100 per day payment. Rather, participants seem to be highly committed to completing the event, perhaps out of commitment to the research team, other participants, and investment in providing advice. We suggest personal communication between the investigators and participants, and clear explanation of the importance of the participant’s role throughout the entire process, played a large role in the retention rate.

A key difference between this deliberative event and previous events is in the method used for recruitment. Previous events have highlighted difficulties in achieving representation of diversity [1,49], whereas the method of recruitment used in the current study was successful in achieving diversity, and retained sufficient flexibility for particular participant categories to be filled purposively. This supports the findings of a recent review which found that “studies systematically seeking to include people from a wide variety of backgrounds by using stratification… tended to be more successful in recruiting diverse voices” [49]. Strong support for the local research institute, and medical research generally, possibly reinforced the success of the method. Younger participants were harder to recruit than older participants, which may be reflective of the shift toward mobile telephone usage or older people and females being more interested in biobanking. Future deliberative democracy events may benefit from including mobile telephone numbers and taking a more diverse approach to promoting the event differently to distinct demographic groups.

## 5. Conclusions and Implications

This event produced recommendations that informed policy makers and biobanking stakeholders responsible for biobank developments in Tasmania. The benefit of this approach over survey methods for capturing public opinion lies in the informed nature of the opinions, derived from the discussion-based format and diverse range of participants. The utility of large-scale prospective epidemiology and biobanking studies is well established, but requires large investment and long-term commitment. Deliberative democracy presents an appropriate vehicle to manage community input into these projects. It has been proposed that deliberative democracy events may be employed whenever broader population participation or support is required. By undertaking a deliberative democracy event prior to the commencement of a study of this nature, areas of trust and concern can be ascertained and monitored throughout the life of the study. Additional deliberative democracy events during the course of the study could be used to examine changing social norms as society evolves and scientific advancements continue to be made. Deliberative democracy events are particularly useful for enhancing participation of those not normally represented, including people from remote, disadvantaged or rural areas. They may also be useful where valuable datasets have been collected under a previous consent model and require consultation to consider current ethical norms for ongoing use.

## Supplementary Files

Supplementary File 1
