# Supplementary material for: Community Engagement for Big Epidemiology: Deliberative Democracy as a Tool
Source: J Pers Med. 2014 Nov 20;4(4):459–74. doi: 10.3390/jpm4040459 (PMC4282883; doi:10.3390/jpm4040459)
Supplement: Supplementary File 1 [file jpm-04-00459-s001.pdf]

Deliberative Community Engagement Telephone *Recruiting SCRIPT*

Hi, my name is [interviewer] and I am calling on behalf of the University of Tasmania. We are not selling anything, but are conducting research on what the public thinks about medical research. As part of this research, we're trying to find 25 Tasmanian residents willing to devote two weekends to a discussion about medical research. The event will take place in April and you would be paid $400 for your time, plus all travel, food and living expenses would be paid for. Would you be interested in hearing more about the event?

[ ] NO → [Skip to rejection reasons]
[ ] YES → Continue

This recruitment has been approved by the Tasmanian Human Research Ethics Committee. Your telephone number was selected by chance from publicly available sources. We are trying to find people from a wide range of backgrounds to participate in the discussion, so I would like to first ask you a few questions to see if you qualify:

- Are you currently a Tasmanian resident?
- Can I verify your age?
- Do you work outside the home?
  - *[If yes:]* What do you do?
  - *[If retired or unemployed, ask for previous employment.]*
- What is the highest level of education you have completed?
- When you are asked on forms to state your ethnicity, what do you say?
- Were you born outside of Australia? If so, where?
- Would you be comfortable speaking publicly in English?
- Is there another language that you are more comfortable speaking?
- Have you ever been diagnosed with a serious disease or health condition?
  - *[If yes:]* Can I ask what it was?
- Has anyone close to you (that is, close friends, partner, family) been diagnosed with a serious disease or health condition?
  - *[If yes:]* Can I ask what it was?

[Callers have demographic tables to complete as they recruit.]

[For under-represented minorities, if the respondent suggests that a family member participate instead, the caller can go ahead and talk to the family member. However, the caller will not solicit this.]

[If the respondent does not meet any of the remaining required categories:]

I am sorry to have troubled you, but we already have people with similar background to yours. Thank you for your time and have a nice day.

[If the respondent fits available categories]: Great! You fit into one of the categories of participants we still need.

We're trying to find people willing to devote two weekends to a discussion about how samples collected for research should be used and stored. The technical term is "Biobanking," which means storing people's blood or other samples to allow for studies of disease or of things that keep people healthy. It is an important area of medical research today, but we don't know how best to protect people whose samples are used, or what concerns people might have. That's why we are interested in talking with people from the community to get their views.

The discussions will happen over two weekends in April on Saturday and Sunday, 6 and 7 April and 13 and 14 April. Because we respect your time, you will be paid $100 dollars for each day you participate – a total of $400. We will pay all expenses, such as meals, accommodation and parking or transportation (if needed), in addition to the $400.

I assure you there are no sales or promotions involved. May I take a few minutes of your time to explain the event a bit more and what we are hoping to achieve?

[ ] NO → [Skip to rejection reasons]
[ ] YES → Continue

The event we plan is called a "Deliberative Community Engagement" because we are asking members of the general public to talk together about Biobanking, consider all the options, and then make recommendations. We are interested in your opinion about how blood and tissue samples <u>should</u> be collected and stored.

You do not need to know anything about the topic to participate, and there will be a series of educational talks to start the event, to make sure everyone

understands the topic. The purpose is to help develop the best possible policies about research with human samples.

While we hope you will find the discussion interesting, you can of course leave at any time. But we've found that many people enjoy the chance to influence policy.

Because talking with members of the general public about how to develop policy has not been done a great deal in Australia, we also plan to study the effectiveness of the Community Engagement itself. We plan to record the discussions and we will also ask you to complete some evaluation forms.

For this Community Engagement to be successful it is very important that we include people who can commit to attending both weekends. Can I include you?

> [ ] NO → Would you be interested in receiving a packet of written material with more detail? You can then have more time to consider this invitation and make a final decision.
>
> > [If No again, skip to rejection reasons]
>
> [ ] YES …. Thank you very much. We will send you additional information by mail.

Can I confirm your name and have your mailing address to send out your package?

Name:
Address:
Postcode:
Telephone:
Email:
Your preferred method of contact:

In the mean time if you have any questions, you may call Dr Rebekah McWhirter on XX XXXX XXXX. Thank you very much and have a great day.

Rejection Reasons:

> To help us understand potential barriers to community engagement, could you let us know why you will not participate?
>
> [recruiters fill out sheet to indicate: unavailable on these dates, lack of interest in event, time commitment is too burdensome, other: ____.]

BiobankProjectTasmania
a community consultation

Assessment of the deliberative events

Welcome to the post-deliberation event survey!

This survey is to help us improve the second weekend and as part of your your assessment of the overall event.

Please write the number that you received from the Biobank Project Tasmania team below. This number will not be linked to your name. It will only be used to compare the pre and post survey responses of all participants.

1. Please rate the event using the following criteria...

	Not at all	A little	Somewhat	Very
How stressful was this event?	○	○	○	○
How enjoyable was this event?	○	○	○	○
How difficult was this event?	○	○	○	○
How worthwhile was this event?	○	○	○	○
How interesting was this event?	○	○	○	○
Overall, were the objectives of the event clear/ understandable?	○	○	○	○
Did you feel heard/listened to during the deliberation?	○	○	○	○
Did you feel included during the deliberation?	○	○	○	○

	Not at all	A little	Somewhat	Very
Did you feel respected during the deliberation?	○	○	○	○
Were the processes that led to the group's recommendations fair?	○	○	○	○
Were the processes that led to the group's recommendations trustworthy?	○	○	○	○
How willing are you to abide by the group's final position even if you personally have a different view?	○	○	○	○

2. Please rate each of the following aspects of the deliberative event.

	Very poor	Somewhat poor	Neutral	Good	Excellent
Booklet I received before the event	○	○	○	○	○
Expert speakers	○	○	○	○	○
Large group facilitator	○	○	○	○	○
Small group facilitator	○	○	○	○	○
Post event surveys	○	○	○	○	○
Agenda setting exercise at the end of the first weekend (where topics were selected for the second weekend)	○	○	○	○	○
My discussions/interactions with other participants	○	○	○	○	○
Other aspects (describe below and rank)					

....

....

3. Did you do any research on biobanks (or related issues) before the first week's sessions?

- ○ Yes
- ○ No

If yes, please briefly describe what kind of research you did and what you found.

....

....

....

....

4. Please indicate your level of agreement with the following statement.

The group's final recommendations addressed all issues considered important by participants.

- ○ Strongly disagree
- ○ Disagree
- ○ I don't know
- ○ Agree
- ○ Strongly agree

5. How likely is it that you would attend an event like this again in the future?

- ○ Not at all likely
- ○ Unlikely
- ○ I don't know
- ○ Likely
- ○ Very likely

6. In your opinion, is it sensible for researchers to rely on a deliberative event like this when trying to develop Australian policy?

○ Yes

○ No

○ Don't know

7. How would you rate this deliberative event overall?

○ Very poor

○ Poor

○ I don't know

○ Good

○ Excellent

8. What words best describe your feelings about this event? (Select as many as you like)

☐ Informative

☐ Challenging

☐ Well-balanced

☐ Boring

☐ Overly simplistic

☐ Organized

☐ Inspiring

☐ Poorly-balanced

☐ Repetitious

☐ Too short

☐ Enjoyable

☐ Stimulating

☐ Disorganized

☐ Overly complex

☐ Too long

☐ Other, please specify...

9. Do you have any final comments for the deliberative team?

These final demographic questions are for statistical purposes only. Your responses to them will be kept strictly confidential and they are all optional.

Question 10

Do you have any children?

- ○ No
- ○ If yes, how many? _______________________

Question 11

What is your marital status?

- ○ Common law relationship
- ○ Married
- ○ Separated
- ○ Divorced
- ○ Widowed
- ○ Single
- ○ Other

Question 12

How frequently do you consume news media (in a newspaper, online, on TV or radio)?

- ○ Less than once a month
- ○ About once a month
- ○ A couple of times a month

- ○ Several times a week
- ○ Daily

Question 13

Which best describes your household income (before tax)?

- ○ Under $20,000
- ○ $21,000-$30,000
- ○ $31,000-$40,000
- ○ $41,000-50,000
- ○ $51,000-60,000
- ○ $61,000-$70,000
- ○ $71,000-$80,000
- ○ $81,000-$90,000
- ○ $91,000-100,000
- ○ Over $100,000
